# Air pollution research: visualization of research activity using density-equalizing mapping and scientometric benchmarking procedures

**DOI:** 10.1186/1745-6673-5-5

**Published:** 2010-04-01

**Authors:** Hanna Zell, David Quarcoo, Cristian Scutaru, Karin Vitzthum, Stefanie Uibel, Norman Schöffel, Stefanie Mache, David A Groneberg, Michael F Spallek

**Affiliations:** 1 Department of Information Science, Institute of Occupational Medicine, Charité-Universitätsmedizin Berlin, Free University Berlin and Humboldt-University Berlin, Thielallee 69-73, 14195 Berlin, Germany; 2 Department of Respiratory Medicine, Hanover Medical School, Carl-Neuberg-Straße 1, 30625 Hanover, Germany

## Abstract

**Background:**

Due to constantly rising air pollution levels as well as an increasing awareness of the hazardousness of air pollutants, new laws and rules have recently been passed. Although there has been a large amount of research on this topic, bibliometric data is still to be collected. Thus this study provides a scientometric approach to the material published on this subject so far.

**Methods:**

For this purpose, data retrieved from the "Web of Science" provided by the Thomson Scientific Institute was analyzed and visualized both with density-equalizing methods and classic data-processing methods such as tables and charts.

**Results:**

For the time span between 1955 and 2006, 26,253 items were listed and related to the topic of air pollution, published by 124 countries in 24 different languages. General citation activity has been constantly increasing since the beginning of the examined period. However, beginning with the year 1991, citation levels have been rising exponentially each year, reaching 39,220 citations in the year 2006. The United States, the UK and Germany were the three most productive countries in the area, with English and German ranked first and second in publishing languages, followed by French. An article published by Dockery, Pope, Xu et al. was both the most cited in total numbers and in average citation rate. J. Schwartz was able to claim the highest total number of citations on his publications, while D.W. Dockery has the highest citation rate per publication. As to the subject areas the items are assigned with, the most item were published in Environmental Sciences, followed by Meteorology & Atmospheric Sciences and Public, Environmental & Occupational Health. Nine out of the ten publishing journals with more than 300 entries dealt with environmental interests and one dealt with epidemiology.

**Conclusions:**

Using the method of density-equalizing mapping and further common data processing procedures, it can be concluded that scientific work concerning air pollution and related topics enjoys unbrokenly growing scientific interest. This can be observed both in publication numbers and in citation activity.

## Background

Air pollution is defined as the emission of particulate toxic elements into the atmosphere by natural or anthropogenic sources [1]. These sources can be further differentiated into either mobile or stationary sources [2]. Anthropogenic air pollution commenced with human's systematic use of fire. Its historical development has been characterized by steadily increasing amounts of total emissions, the invention of new sources of pollution emission as well as the emission of pollutants that had not formerly been emitted by man-made sources. So far, this development has had the greatest impact on the air quality of so-called Mega-Cities (cities with over 10,000,000 inhabitants). Today the major sources of man-made air pollution are motorized street traffic (especially exhaust gases and tire abrasion), the burning of fuels, and larger factory emissions. Depending on the pollutant particles' size, they can be carried for distances of several thousand miles. With decreasing diameter, they are able to infiltrate finer lung structures [3].

The World Health Organization (WHO) estimates 2.4 million fatalities due to air pollution each year [4]. Since the breathing of polluted air may have severe health effects such as asthma, COPD or increased cardiovascular risks [5,6], most countries have strengthened laws to control the air quality in the past decade. Further, as polluted air is considered a super-regional problem, international conferences have recently developed different ways to improve and assure air quality employing global strategic perspectives [7,8].

Despite such enormous scientific and legislative efforts to measure and improve air quality levels, many people are still exposed to hazardously polluted breathing air on a daily basis [9,10]. Furthermore, there are currently no complete bibliometric analyses available on this topic.

The present scientometric compilation and analysis is intended to identify current scientific efforts with regard to air pollution, as well as to highlight research gaps requiring further attention.

## Methods

All analyzed data were retrieved from Thomson Scientific's online-database "Web of Science". To provide a comparison data was also collected in some cases from "PubMed", the online database of the U.S. National Library of Medicine.

For the query, the terms "air pollution" and "air quality" were connected with the Boolean operator "OR" and entered in the search field "General Search".

The time frame was limited to the period between 1955 and 2006. For this purpose, the "Change Limits and Settings" function was adjusted initially and the query was made under this presetting. The limitation was performed since the years 2007-2009 were not finalized in the databases completely.

Results were also analyzed by means of citation. Therefore, the feature "Citation Report" was used to calculate the citation rate of both authors and citations per year of citation. To calculate the citation rates of the singular authors, results were first sorted by author. Afterwards, the ten most productive authors' publications were put under citation analysis. The average citation rate is the quotient of the total citation number divided by the publications listed for the author in question.

The "Web of Science" database provides several tools to analyze entries according to specified parameters [11]. The data set was analyzed by means of publication country, publication year, publishing author, publishing journals and published document type. Multiple distributions led to higher publication numbers when adding up results after analysis; for example, when a super regional publication was distributed to several countries. A common data processing program was used to display the results in tables, charts and diagrams.

Software using the method of density-equalizing mapping was employed to determine international correlations. This method resizes countries proportionally according to a predefined variable. In this study, the territory with the highest number of publications is depicted largest on the associated map. The basic principle was developed by Gastner and Newman [12].

## Results

### Total number of published items

**Figure 1:**
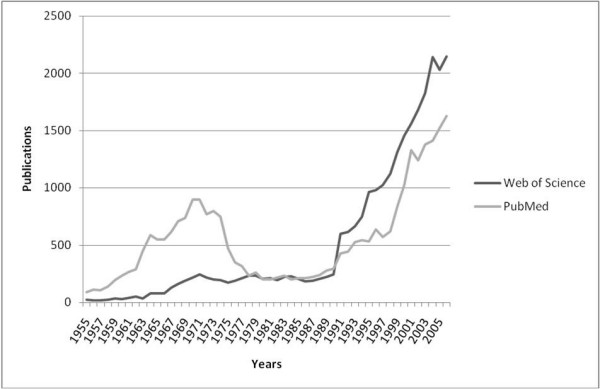
**Publications related to the topic air pollution 1955-2006**. Comparison of results in „Web of Science" and „PubMed".

The overall number of items listed in the database served as a measure of both public interest and scientific productivity concerning the topic of air pollution. The comparison of results in "Web of Science" (26,253) and "PubMed" (28,565) indicated some differences. Entries in the "Web of Science" displayed a comparatively constant number over 25 years (1966-1990), following lower numbers in the prior decade (1955-1965). "PubMed" results, however, differed: Beginning with the year 1957 publication numbers increased up to a preliminary maximum in 1971. Subsequently, decreasing publication numbers equalized that of the "Web of Science" results again in 1978. After a period of relatively constant publication quantities (1978-1990), both databases showed an exponential increase in yearly published items. Despite recent points of nominal decline (2002 in "PubMed", 2005 in "Web of Science") the upward tendency remained consistent through 2006 (fig. 1).

### Citations per year

**Figure 2:**
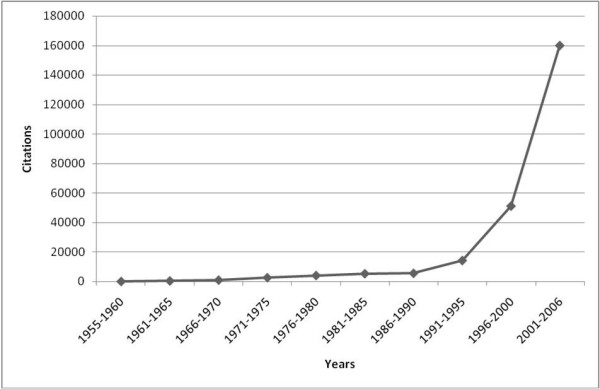
**Citation per Year**. Citations displayed in five-years-intervals.

Regarding the total citations per year (i.e. the overall citation activity for all entries), the numbers show a development similar to publication data. However, the increase since 1991 is marginally sharper than that of the publication numbers (fig. 2).

### Analysis of origin/language

**Figure 3:**
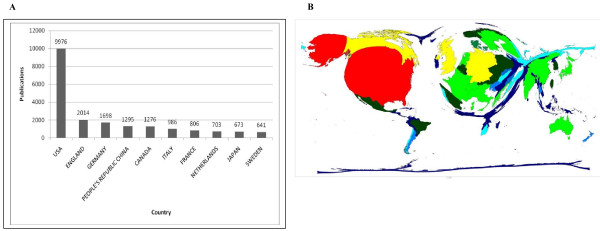
**Publication numbers**. (a) Publications in totalnumbers, sorted by countries (b) Publications sorted by countries put into relation to each other (density-equalized).

Usage of the analysis tool "Countries/Territories" indicates that the USA holds the most publications by far. The UK and Germany ranked a distant second and third. The ten most publishing countries produced 76.44% of all the entries in the inquiry time frame. (fig. 3a) Density-equalizing mapping demonstrates a huge contingent of publications provided by only a few countries' researchers (fig. 3b).

**Figure 4:**
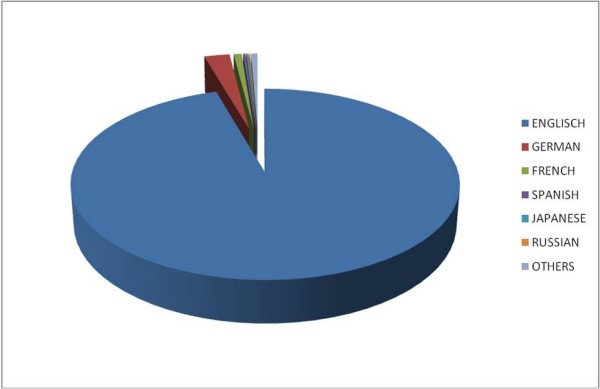
**Publication languages**. Languages used in publications.

As to the analysis by language, results showed a division consistent with the results obtained in the "country" analysis. The percentage of items published in English (96%) was much higher than the fraction published by Anglophone countries (fig. 4).

### Citation characteristics

#### Average citation rate (countries)

**Figure 5:**
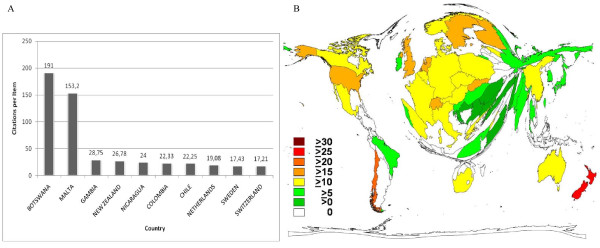
**Citations per country**. (a) Average citations per publications. (b) Average citations per publications (density-equalized). Threshold of 30 publications per country.

To obtain the average citation rate of single countries, the total number of citations for publications originated in each country was divided by the number of the said publications. In conclusion, Botswana achieved the highest rate with 191 citation/item, followed by Malta with 153.2/item. No other country achieved a citation rate higher than 30/item (fig. 5a).

Inclusion of a threshold of at least 30 publication and density-equalizing calculations leads to a cartogram shown in figure 5b.

#### Average citation rate (publications and authors)

The average citation rate was calculated both for the most productive authors and the most cited publications. While the single author's citations had to be divided by his number of publications, the publications' total number of citation was divided by the number of years of citation activity for this item ("citations per year").

As for the publications' total citations, the most cited article was written by Dockery, DW, Pope, CA, Xu, XP et al. and published in the New England Journal of Medicine in 1993. The ten most cited entries in terms of total citation are shown in Table 1, indicating author, title, publishing journal, publication date and the total citation number. Table 2 shows the ten items with the highest citation rate per year.

**Table 1:** Ten most cited articles; citations given in total numbers

Author	Title	Publishing Journal	Publication Date	Total Number of Citations
Dockery, Pope, Xu	An Association Between Air-Pollution and Mortality in 6 United-States Cities	New England Journal of Medicine	1993	1961
Beasley, Keil, von Mutius	Worldwide Variation in Symptoms of Asthma, Allergic Rhinoconjunctivitis, and Atopic Excema: ISAAC	Lancet	1998	1301
McGinnis, Foege	Actual Causes of Death in the United States	Jama-Journal of the American Medical Association	1993	1273
Guenther, Hewitt, Erickson	A Global Model of Natural Volatile Organic-Compound Emissions	Journal of Geophysical Research-Atmospheres	1995	1032
Pope, Thun, Namboodiri	Particulate Air Pollution as a Predictor of Mortality in a Prospective Study of US Adults	American Journal of Respiratory and Critical Care Medicine	1995	950
Murray, Lopez	Global Mortality, Disability and the Contribution of Risk Factors: Global Burden of Disease Study	Lancet	1997	864
Atkinson, Baulch, Cox	Evaluated Kinetic and Photochemical Data for Atmospheric Chemistry Supplement IV-IUPAC Subcommitee on Gas Kinetic Data Evaluation for Atmospheric Chemistry	Journal of Physical and Chemical Reference Data	1992	813
Pope, Burnett, Thun	Lung Cancer, Cardiopulmonary Mortality and Long-Term Exposure to Fine Particulate Air Pollution	Jama-Journal of the American Medical Association	2002	778
Dockery, Pope	Acute Respiratory Effects of Particulate Air Pollution	Annual Review of Public Health	1994	776
Samet, Dominici, Curriero	Fine Particulate Air Pollution and Mortality in 20 US Cities, 1987-1994	New England Journal of Medicine	2000	613

**Table 2:** Ten most cited articles; citations per year

Author	Title	Publishing Journal	Publication Date	Citations per Year
Dockery, Pope, Xu	An Association Between Air-Pollution and Mortality in 6 United-States Cities	New England Journal of Medicine	1993	122,56
Beasley, Keil, von Mutius	Worldwide Variation in Symptoms of Asthma, Allergic Rhinoconjunctivitis, and atopic Excema: ISAAC	Lancet	1998	118,27
Pope, Burnett, Thun	Lung Cancer, Cardiopulmonary Mortality and Long-Term Exposure to Fine Particulate Air Pollution	Jama-Journal of the American Medical Association	2002	111,14
Mokdad, Marks, Stroup	Actual Causes of Death in the United States 2000	Jama-Journal of the American Medical Association	2004	108,60
McGinnis, Foege	Actual Causes of Death in the United States	Jama-Journal of the American Medical Association	1993	79,56
Guenther, Hewitt, Erickson	A Global Model of Natural Volatile Organic-Compound Emissions	Journal of Geophysical Research-Atmospheres	1995	73,71
Murray, Lopez	Global Mortality, Disability and the Contribution of Risk Factors: Global Burden of Disease Study	Lancet	1997	72,00
Samet, Dominici, Curriero	Fine Particulate Air Pollution and Mortality in 20 US Cities, 1987-1994	New England Journal of Medicine	2000	68,11
Pope, Thun, Namboodi-ri	Particulate Air Pollution as a Predictor of Mortality in a Prospective Study of US Adults	American Journal of Respiratory and Critical Care Medicine	1995	67,86
Ezzati, Lopez, Rogers	Selected Major Risk Factors And Global And Regional Burden of Disease	Lancet	2002	62,14

**Figure 6:**
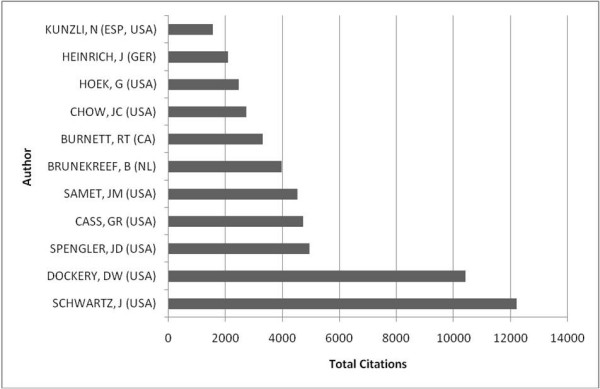
**Citations per author/total**. Citations of the ten most productive authors; citations given in total numbers.

**Figure 7:**
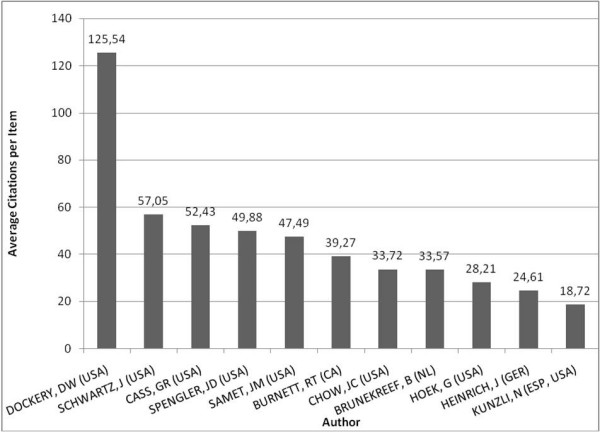
**Citations per author/item**. Citations of the ten most productive authors; citations given in citations per item.

Examining the ten most productive authors' publications, J. Schwartz was cited most often in terms of total numbers (fig. 6), while D.W. Dockery achieved the highest citation rate ("citations per item") (fig. 7).

### Analysis of assigned subject areas

**Figure 8:**
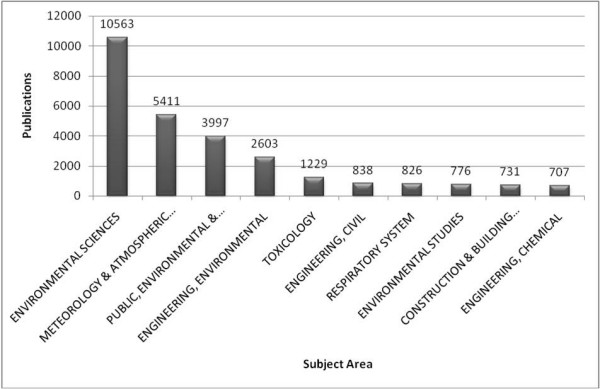
**Subject areas**. Publications' assignment to subjects areas; publications given in total numbers.

Analysis of the subject areas assigned to the publications revealed only two involved medical issues in the first ten areas. The other eight areas dealt either with environmental questions or with matters related to engineering (fig. 8).

### Publication type

**Figure 9:**
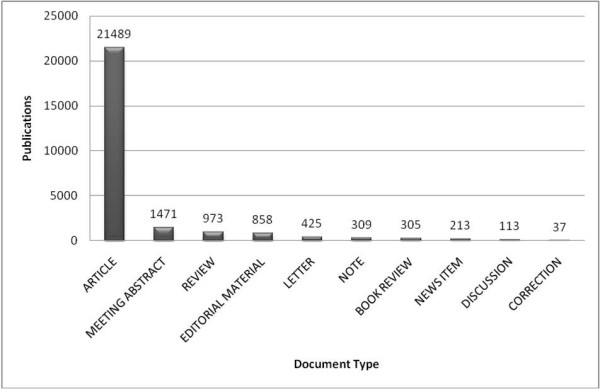
**Document type**. Publications displayed by published document type; publications given in total numbers.

The most commonly published document type was the article, followed by the meeting abstract. The ten most frequently used document types made up for 99.77% of all publications (fig. 9).

### Publishing journals

**Figure 10:**
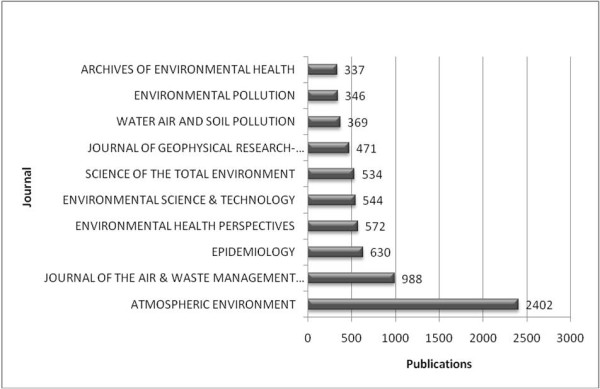
**Publications per journal**. Number of publications in the ten most productive journals; publications given in total numbers.

Out of the ten most publishing journals, nine dealt with environmental subject matters and one was dedicated to epidemiology (fig. 10). Items published in those journals added up to 27.40% of the entire amount of results.

Despite the fact that a similar search request in the medical database MEDLINE generated more results (28,565) than the one in "Web of Science", the entries were concerned more with environmental and technical interests than with medical subject matters. The first journal solely dedicated to toxicology (Inhalation Toxicology) was ranked thirteenth.

## Discussion

Air pollution has always been a subject of public concern. Particularly from the mid-twentieth century onwards, there has been a growing societal impetus to curtail and counteract the hazardous effects of air pollution. In recent decades, as anthropogenic air pollution has reached historically high levels, international public and scientific interest has intensified towards this topic. While natural, stationary emission cannot be significantly influenced, a major focus has been given to changes in man-made pollutant emissions. Heretofore, there has been no comprehensive analysis of data available on this topic. The present study sought to provide bibliometric data on research activity related to the subject air pollution, analyzed and displayed with both common and innovative data processing methods such as density-equalizing calculation. The examined time frame was limited to the period after 1955, since global research activity was relatively low before that date. The year of 2007 was excluded from the analysis because of incomplete data.

Mirroring public and scientific interest, general research activity on the topic of air pollution has increased steadily since the beginning of the analyzed period. However, a remarkable boost in both publication and citation activity can be observed at the beginning of the 1990s. On the one hand, this can be attributed to the availability of public internet access [13]. Before the early 1990s, access to such research data was restricted primarily to scientific institutions, larger companies and governmental organizations. On the other hand, authors' abstracts for the publications were only available since the year 1991 and authors of foreign languages were especially encouraged to write in English [14]. Thus, the major increase in citation activity observed since the beginning of the 1990s may be explained by an eased availability of contents both in the short form of the abstract as well as making summaries of publications in different languages available in English [15]. The increase in publication activity may be related to the easier access to the internet as a publication provider. Additionally, the massive simplification of data disposability in the shape of changing from paper to electronic devices might have encouraged authors to publish more of their findings.

Regarding the origin of publications, US researchers contribute by far the largest amount of scientific output related to air pollution. The second ranking of Great Britain, the third ranking of Germany and especially the People's Republic of China in fourth position is notable as well. Mega-Cities are defined as urban agglomeration areas with more than 10,000,000 inhabitants. They are characterized by social challenges such as poverty and crime but above all for producing massive environmental burdens especially in the field of air quality [16]. Considering the fact that three of the world's 26 mega-cities are located in China, four in directly neighbouring countries and another four in nearby countries (i.e. Japan, South-Korea and Bangladesh), it may be legitimate to contribute the large interest in air quality matters partly to those circumstances. Altogether it can be said that a minority of the world's countries contributes the majority of general research activity, as shown by density-equalizing methods [17]. However, the number of highly polluted areas (such as Mega-Cities) as well as the total amount of pollutants emitted should be connected to the high research effort of few countries.

English appears as the most common language in scientific releases; a finding that is concordant with papers' distribution to countries. While German plays a comparatively major role too, Chinese cannot be found among the most frequently used languages. This disproportion might lead to the assumption that English is used more commonly among Chinese researchers compared to European scientific publications. While French ranked third among the publications' languages, France's number of publications ranked seventh - past Italy and China whose languages do not appear at all among the most common ones. Russian also appears among the most frequently used languages, though Russia's publications ranked 27th in the international list. These facts could either be ascribed to political conditions and convictions, or to closer or broader affinity of languages. It is possible that states belonging to the former USSR still use Russian as their scientific language after the independency from the Russian government. It is also possible that Baltic and Eastern European States tend to rely more on research by their former authority (i.e. Russia) than Western European or American researchers and therefore use rather Russian than English in publications. Additionally, Slavic languages are linguistically closer related to Russian than to English. Older researchers might also have difficulties writing in a new language after using Russian for several decades.

While the total amount of publications was used as a distinguishing mark for research quantity, the average citation rate was introduced to evaluate the single nations' research quality [18]. In this regard Botswana ranked first with 191 citations per items followed closely by Malta with 153.2/item. Gambia ranking third could only unify 28.75 citations on every item published. As authors working in Botswana only published seven items in the whole time period investigated, the high citation rate appears exceptional. Malta unifying 1,532 citations on ten items is rather inconsequential as well. Closer attention to the results from the countries in question revealed that a world-wide asthma-study on children published in 1998 was distributed to all the 56 countries participating [19]. With 1,301 citations on that single item, it has a larger impact on the average citation rate, the lower the total publication number. Therefore the uncommonly high average citation rates for both Botswana and Malta must be credited with this international, highly-attended study. To avoid disproportionately high citation rates due to low total publication numbers, a borderline was drawn at ten publications. Given this condition and additionally taking the aforesaid study out of consideration, the Netherlands moved up to the first position, showing 19.08 citations per item followed by Sweden (17.43) and Switzerland (17.21).

Among the most cited articles, the aforementioned international asthma study is itself exceeded by an article by Dockery et al. in the New England Journal of Medicine, on the association between air pollution and mortality in six US cities [20]. This is one of the first studies pointing out the association between air pollution by particulates and sulfates and increased death rates due to pulmonary causes, deducting additional risks such as smoking beforehand.

As an author, D.W. Dockery features the highest average citation rate (125.54). However, in total citations, Dockery is only second to J. Schwartz.

With regard to "subject areas", the environmental sciences have produced the most results so far on the topic of air pollution, followed by the atmospheric sciences. Noticing that among the ten leading research fields there are only three concerned with health aspects, it can be said there is a rather significant lack of medical research on this subject. Given an estimated 2.4 million deaths yearly due to air pollution, it is rather surprising that medical research has lagged by this degree. Considering all the severe consequences polluted air has on public health, on international health conditions, and health care costs, it is justified to point out this obvious research gap, and recommend further scientific efforts in medicine in the future.

Since most of the publications are articles, it can be said that most of the scientific endeavours have been embossed by initiatives rather than by efforts in analysis and description of statistical coherences.

Among the ten most publishing journals, nine are occupied with environmental issues and one with epidemiology. This again demonstrates the existing deficit in medical interest.

However, it may be that medical interest in the field of air pollution and its consequences for human health may have developed only recently. Despite the fact that polluted air has always been a major threat on human health, there has not been any major supra-regional effort to change the polluting behaviour in the past 20 years. Although people have been suffering from polluted air for a along time, beginning several centuries ago, there has been no substantiated evidence for the connection between polluted air and deaths due to pulmonary diseases. New conclusions about coherences between certain air pollutants and their impact on public health have only been drawn recently, and therefore it is entirely possible that the medical research efforts will eventually catch up to and even exceed the scientific work already done in environmental fields.

## Conclusions

Hereby given the first comprehensive analysis of scientometric data on the subject of air pollution, it can be said that scientific interest in this topic has steadily increased to the present day. However, there is to be noted a major research gap in terms of medical analysis. The major contingent of data originates from the USA and an even larger amount is written in the English language. Considering research quality as measured in terms of average citation, less productive countries such as the Netherlands and Sweden show the best results. A generally larger effort towards medical research into air pollution is strongly indicated at this time.

## Competing interests

The authors declare that they have no competing interests.

## Authors' contributions

HZ carried out the bibliometric investigations, participated in analyzing results and drafted the manuscript. CS participated in data research and performed the scientometric analysis. DQ, NS, SM participated in the design of the study. KV and SU carried out pilot data research. DAG participated in its design and data analyses. MS conceived of the study, and participated in its design and coordination and helped to draft the manuscript. All authors read and approved the final manuscript.
